# Late Presentation of Pancreatic Pseudocyst in a Child

**DOI:** 10.1097/PG9.0000000000000010

**Published:** 2020-09-28

**Authors:** Lova Hasina Rajaonarison Ny Ony Narindra, Biendoux Tombomiadantsoa, Francis Allen Hunald, Ahmad Ahmad

**Affiliations:** From the * Medical Imaging Center, CHU HJRA Antananarivo, Antananarivo, Madagascar; † Paediatric Surgery Center, CHU JRA Antananarivo, Antananarivo, Madagascar.

Pancreatic pseudocysts are rare in pediatrics (1). They can be asymptomatic or alarming by their large size and its complications. We report a large pancreatic pseudocyst in a 13-year-old girl diagnosed on computed tomography exam 8 months after abdominal trauma. The child underwent an internal cystogastric bypass and the pathology examination confirmed the diagnosis. The objective of this observation is to describe the diagnostic aspects and therapeutic management given the data in the literature.

## OBSERVATION

**FIGURE 1.**
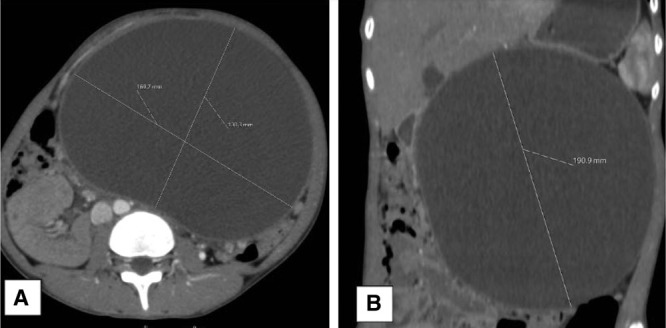
Abdominal CT scan with contrast media at the arterial phase, in axial section (A) and coronal reconstruction (B) showing a large retroperitoneal cystic mass related to a pancreatic pseudocyst. Note the right side repression of the retroperitoneal vessels.

**FIGURE 2.**
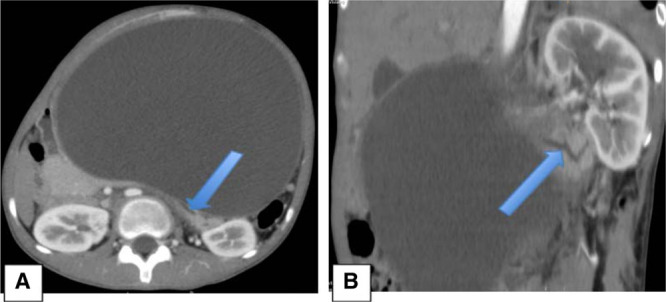
Abdominopelvic CT scan with contrast media in arterial phase, in axial section (A) and oblique coronal reconstruction (B) showing the large cystic mass with dilation of the main pancreatic duct of the caudal portion (arrows).

**FIGURE 3.**
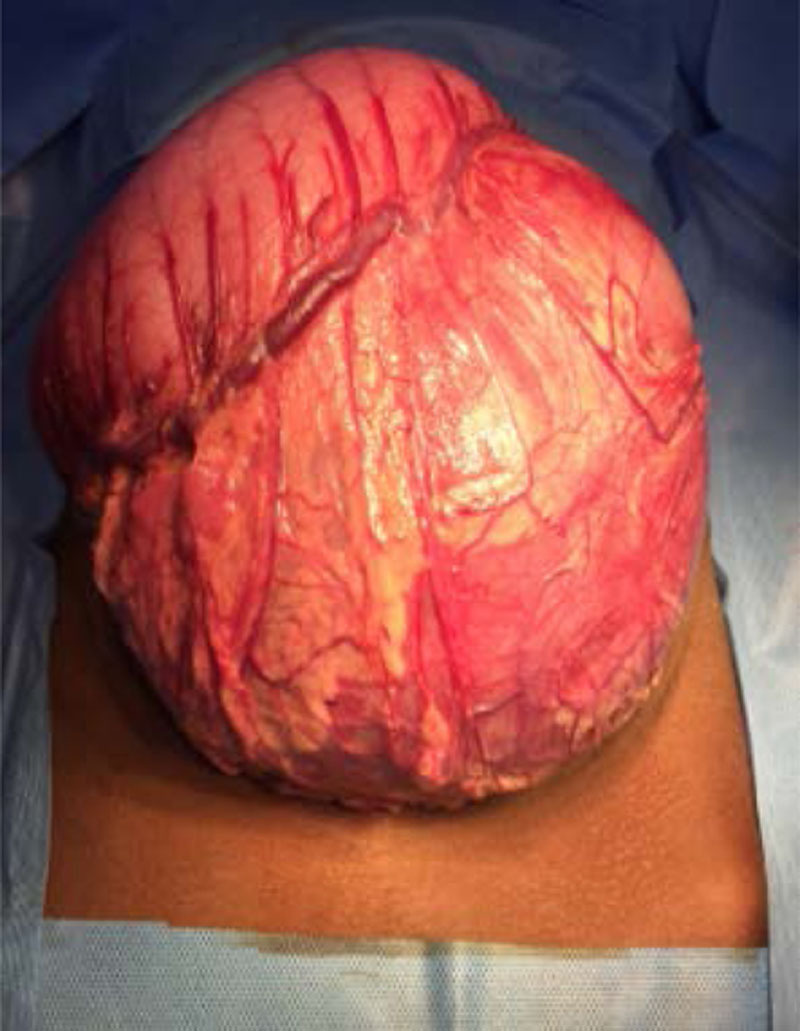
Intraoperative photograph of the pancreatic pseudocyst.

A 13-year-old girl was admitted for chronic epigastric pain with a distended abdomen. There was a history of abdominal trauma by beef kick 8 months earlier requiring 4 days of hospitalization but without specific exploration. There was no particular personal or familial history including pancreatitis or hyperlipidemia. The clinical examination showed a distended abdomen with collateral venous circulation, dull to percussion with the sensation of a soft mass. There were no clinical and biological inflammatory signs. Standard tests as blood sugar, creatinine, blood and urine ionogram, amylasemia, and lipasemia were normal at the time of diagnosis. The tumor markers as lactate dehydrogenase, alpha-fetoprotein, and beta-human chorionic gonadotropin were normal. Abdominal ultrasound revealed a large anechoic cystic mass not clearly attached. The abdominopelvic computed tomography exam confirmed the bulky cystic mass with homogeneous hypodense content surrounded by a thin and regular wall measuring 190 × 172 × 125 mm in size at the anterior face of the body of the pancreas (Fig. 1) with dilation of the main pancreatic duct of the caudal portion (Fig. 2). A laparotomy (Fig. 3) for internal cystogastric bypass was performed after the bacteriological and chemical puncture of the fluid and biopsy of the cyst wall which confirmed a pancreatic pseudocyst on anatomopathological examination. The postoperative follow-up was marked by the appearance of fever on the fifth day with evidence of Enterobacter spp in blood culture motivating the change in antibiotic therapy. Apyrexia was again obtained on the 10th day and the child was discharged from the hospital on the 12th day. The 3-month ultrasound check was without abnormality.

## DISCUSSION

Pancreatic pseudocysts are rare benign conditions (1,2). It is a liquid collection made of pancreatic juice, of possible necrotic or hemorrhagic debris surrounded by fibrous tissue devoid of the epithelium (3) which can be localized in intra- or extra-pancreatic. If the pancreatic pseudocysts can be of exceptional congenital origin (2), the frequent etiologies are dominated by acute pancreatitis and pancreatic trauma (4,5). There is a male tendency for theses damages in the small series of cases reported. Indeed, the 7 cases of Cherrabi et al (5) are all boys while Makin et al (4) has reported 5 male cases out of a series of 7; Bolia et al (6) collected 43 boys out of a series of 58 children. The median age of children is around 10 years at diagnosis. The clinical signs are dominated by chronic epigastric pain and abdominal mass but also signs related to the complications. The pseudocyst is part of the natural course of pancreatic trauma or acute pancreatitis. Thus, out of a multicentric series of 100 children suffering from pancreatic trauma collected in 22 pediatric trauma centers in America, Canada, and the United Kingdom for 5 years (2010–2015), 64% have developed acute peripancreatic collections and 36% (27 children) among them have developed a pancreatic pseudocyst (7). Similarly, out of a monocentric series of 58 cases of acute pediatric pancreatitis, of idiopathic and traumatic origin, collected for 10 years (2001–2011), 38% developed a pancreatic pseudocyst (6). The time to onset of the pseudocyst after the episode of acute pancreatitis or trauma is variable between 4 and 6 weeks but can be very late (1). In our patient, the diagnosis was late, 8 months after the causal trauma. Generally, it is a blunt trauma with epigastric reception even if our case was a minimal abdominal trauma. The size of the pseudocysts is very variable depending on the series, 3–14.4 cm for Bolia et al (6), 2.2–33 cm for Rosenfeld et al (7), and 19 × 17.2 × 12.5 cm for our patient. The cyst is considered to be large beyond 7 cm in large diameter and giant from 20 cm (7). These large pseudocysts take a long time to subside and require drainage (7) even if an 18 cm pseudocyst has spontaneously resorbed among the 4 cases reported by Nouira et al (1). However, from a diameter of 5 cm, the rate of spontaneous regression is low and the risk of complications major (8). The discovery of a pseudocyst should trigger close clinical, biological, and radiological monitoring to detect complications if drainage is not indicated. Among these complications, we can mention secondary infection, hemorrhage or rupture, and vascular or biliary or digestive compression (1). Abdominal ultrasound can help characterize the lesion if itis not large. Good preparation before the examination with a fasting patient from 6 to 8 hours (3 hours for newborns) combined with administration of liquid in the stomach during the examination would improve the sensitivity of the ultrasound in the supine or right lateral position (9). When the pseudocyst is bulky, an abdominal CT scan is indicated to better describe its relations, the associated signs as well as the complications. However, the CT scan is irradiating and limited in the analysis of the contents of the pseudocyst and possible communication with the pancreatic ducts. Thus, biliopancreatic magnetic resonance imaging could be useful to unmask the communication of the pseudocyst with the pancreatic ducts and to discern necrotic debris or hematic components in the pseudocyst (5,9). The differential diagnosis can however be discussed between a mesenteric cyst or a digestive duplication in this localization in these pediatric populations (2) but the clinical context with the history of abdominal trauma as well as the pancreatic relations of the mass associated, in our case, with the dilation of the main pancreatic duct allow to evoke the diagnosis before the anatomopathological confirmation. The therapeutic management depends above all on the symptomatology and the size of the pancreatic pseudocysts. Conservative treatment with active clinical, biological, and radiological follow-up is recommended especially for pseudocysts less than 5 cm in diameter and asymptomatic (6,7). Large pseudocysts require drainage since they do not have a tendency to spontaneous resorption and are fraught with complications (5,7). Percutaneous drainage under radiological guidance seems to be a reliable therapeutic technique to be performed in first intention (5). There is no significant difference between endoscopic and surgical treatment in terms of efficacy (5,7). Transpapillary drainage can be achieved and appears to be most reliable if it is possible. Laparoscopic cystogastrostomy can be performed if the endoscopic or radiological technique is not available (5,6). In the absence of these techniques, internal cystogastric bypass by surgical laparotomy remains indicated (5). Evolution is generally good with all these techniques and hospital discharge is around the eighth day especially for the endoscopic technique.

## CONCLUSION

The child’s pancreatic pseudocyst can be large and difficult to diagnose. The history of abdominal trauma and the dilation of the main pancreatic duct communicating with the cyst are decisive clinical and morphological factors. The cystogastric bypass by laparotomy remains the appropriate treatment in the absence of endoscopic and radiological treatment.
